# A Cost Benefit Analysis of an Active Travel Intervention with Health and Carbon Emission Reduction Benefits

**DOI:** 10.3390/ijerph15050962

**Published:** 2018-05-11

**Authors:** Ralph Chapman, Michael Keall, Philippa Howden-Chapman, Mark Grams, Karen Witten, Edward Randal, Alistair Woodward

**Affiliations:** 1 Environmental Studies Programme, School of Geography, Environment and Earth Sciences, Victoria University of Wellington, Wellington 6140, New Zealand; mawkie@gmail.com; 2 NZ Centre for Sustainable Cities, Wellington 6242, New Zealand; Michael.keall@otago.ac.nz (M.K.); philippa.howden-chapman@otago.ac.nz (P.H.-C.); karen.witten@massey.ac.nz (K.W.); edward.randal@otago.ac.nz (E.R.); a.woodward@auckland.ac.nz (A.W.); 3 Department of Public Health, University of Otago, Wellington 6242, New Zealand; 4 Massey University, SHORE and Whariki Research Centre, Auckland 1141, New Zealand; 5 School of Population Health, University of Auckland, Auckland 1142, New Zealand

**Keywords:** cost benefit analysis, active travel, health, climate change, co-benefit, discounting, ACTIVE, ITHIM

## Abstract

Active travel (walking and cycling) is beneficial for people’s health and has many co-benefits, such as reducing motor vehicle congestion and pollution in urban areas. There have been few robust evaluations of active travel, and very few studies have valued health and emissions outcomes. The ACTIVE before-and-after quasi-experimental study estimated the net benefits of health and other outcomes from New Zealand’s Model Communities Programme using an empirical analysis comparing two intervention cities with two control cities. The Programme funded investment in cycle paths, other walking and cycling facilities, cycle parking, ‘shared spaces’, media campaigns and events, such as ‘Share the Road’, and cycle-skills training. Using the modified Integrated Transport and Health Impacts Model, the Programme’s net economic benefits were estimated from the changes in use of active travel modes. Annual benefits for health in the intervention cities were estimated at 34.4 disability-adjusted life years (DALYs) and two lives saved due to reductions in cardiac disease, diabetes, cancer, and respiratory disease. Reductions in transport-related carbon emissions were also estimated and valued. Using a discount rate of 3.5%, the estimated benefit/cost ratio was 11:1 and was robust to sensitivity testing. It is concluded that when concerted investment is made in active travel in a city, there is likely to be a measurable, positive return on investment.

## 1. Introduction

It is increasingly being acknowledged that active travel (mainly walking and cycling) is beneficial for people’s health, for reducing carbon emissions, and for the smooth functioning of urban areas. From a health viewpoint, physical activity, such as that achieved by active travel, has clear and quantified physical and mental health benefits [1]. From an urban planning and design viewpoint, active travel contributes to a sense of vitality and social cohesion in towns and cities—walkers and cyclists enliven the city [2]—as well as reducing motor vehicle congestion. Many cities have been working to create high-quality local neighbourhoods with compact, mixed-use developments well served by both public transport and walking/cycling facilities, with an associated aim of reducing the need for car travel and reducing emissions [3,4]. This is partly motivated by a desire to have high quality neighbourhoods, which attract prosperous citizens who want more sustainable and less car-dependent lifestyles, together with an awareness that active and public transport are cost-efficient and sustainable means of urban travel [5].

There are wide variations across countries in active travel and car ownership levels, with high car use reducing opportunities for active journeys in many cities. Cultural, social, and historical factors appear to lie behind the high levels of active travel in northern Europe, contrasting with the low levels in Anglophone countries, especially US, Canada, Australia, and New Zealand. For example, the cycling share of trips in Amsterdam was 37% in 2005 [6] while in Auckland the share for walking and cycling together was only 17% in 2013 [7]. Urban form interacts with transport infrastructure to reinforce these different patterns; for example, a car-intensive transport system tends to become congested and spread out, leading to pressure for more road investment and urban sprawl [8], while making active transport less safe, attractive, and practical [6] (p. S121). In short, active travel is far from widely supported in countries such as New Zealand, and faces many obstacles to its further take-up, not least of which is uncertainty about how its benefits compare to its costs.

The paper is structured as follows. In this Section, we argue that there have been few robust evaluations of active travel; that there remains considerable debate about the estimation of benefits; and that very few studies place a value on health and emissions outcomes and link them back to active transport investment in a rigorous cost-benefit analysis (CBA). In Section 2, we first outline briefly in Section 2.1, the ‘ACTIVE’ before-and-after quasi-experimental study [9,10], which provided our empirical data on the effectiveness of New Zealand’s Model Communities Programme. This intervention programme comprised mainly infrastructure improvements but also education and promotion. We explain in Section 2.2 how, taking these empirical results, we moved to a CBA of the programme’s health and other outcomes. Section 3 sets out the results and Section 4 and Section 5 a discussion and conclusion.

### What Is Known about the Economic Evaluation of Active Travel Interventions?

There is ongoing discussion about what are the most important outcomes of active travel interventions to consider and how to assess them [11,12], with CBA frameworks strongly influencing intervention programme decisions [13] even though CBA deals poorly or not at all with considerations such as equity and long-term land use impacts [14,15]. In New Zealand, CBAs retain considerable significance in the transport investment setting despite a reduced role over the past decade [13,16]. Health outcomes are not always captured in assessments of active travel interventions, but when they are, they tend to dominate the range of benefits [17] (but see Powell et al. [18] discussed below). A number of studies have emerged since the World Health Organization deplored the ‘serious lack of cost-effectiveness studies for all types of environmental health interventions’ [19].

Krizek [20] noted that most active transport evaluations fall far short of the ideal research design, which would at least include before-and-after measurements of a treatment and a control group. Götschi et al. underlined the need to link investments and health benefits in order to provide evidence to ‘achieve shifts towards healthier travel patterns’ [1] (p. 46).

Estimating the *costs* involved in CBAs of active travel is generally straightforward, but there is ongoing debate about benefit estimation. Evaluation of benefits in studies such as the present one has two important features. Firstly, regulatory or investment responsibilities (which may rest, for instance, on transport/infrastructure agencies) and benefits often span different sectors (including the health sector) and include non-health outcomes, such as time saving, increased amenity, and so on, making data collection and consistent valuation complex. Secondly, many of the benefits (such as better health and reduced carbon emissions) are long-term in nature, so that a high or even moderate discount rate (the rate at which future values are translated into today’s values) can significantly reduce the intervention’s estimated net present value [19].

In recent years, economic analyses focusing on active transport infrastructure or other active transport interventions have included measurement of health effects together with more clearly outlined methodology and assumptions. A 2016 systematic review of economic analyses of active transport interventions [21] found 36 credible studies; 23 were studies of proposed or hypothetical interventions, and 13 were studies of implemented interventions. The review concluded that, overall, the quality of the evidence was weak. This conclusion is consistent with an earlier review by Powell et al. [18], who noted limitations in design of even the best studies they examined: for example, none used a control group to establish effectiveness by capturing changes in outcomes that may have occurred anyway or evaluated carbon savings, which have important environmental and economic implications.

Among the more robust studies mentioned in these reviews, benefit-cost ratios (BCRs) were usually between 1 and 10:1, but high study heterogeneity means it is difficult to generalize. For example, a study of walking and cycling network investment initiatives in Norway [11] found that, although BCRs were uncertain due to the range of assumptions necessary in estimation (including around induced network patronage), they were likely in the range 3:1 to 14:1. Another study [22] suggested a BCR for *mortality* benefits of the Cycling Demonstration Town programme of around 2.6:1, although this result was limited by being based on recall of cycling in a ‘typical week in the last year’ and a restricted scope of benefits.

A recent Copenhagen study [17] valued a range of benefits of cycling which are not conventionally quantified, including savings in carbon, noise, congestion, and road deterioration, and branding/tourism benefits. They confirmed that by far the largest social benefits from cycling were health gains, followed by the congestion and noise savings from reduced car traffic, with climate benefits having a magnitude similar to air pollution benefits. On the disbenefit side, the net social costs of crash injuries associated with cycling rather than car use offset less than 20% of the health benefits. The Copenhagen study’s conclusions on health (and injury) impacts are broadly consistent with a study of infrastructure investments in the U.K. designed to encourage walking and cycling [14], which found that the dominant benefit was increased physical activity (a health/mortality benefit derived mainly from the provision of cycle parking), and those of a 2016 simulation study [23] of the health and other effects of transport policies in six European cities.

The importance of health outcomes has given impetus to the development of frameworks, such as the ‘Integrated Transport and Health Impacts Model’ (ITHIM), that build on the work of Woodcock et al. [24] and Rutter et al. [25], who developed a health economic assessment tool (‘HEAT’) for estimating *mortality* benefits of cycling interventions. ITHIM’s principal sub-models are for physical activity, air pollution exposure, and injury [26], and outputs are expressed as annual reductions of deaths and DALYs (disability-adjusted life years, years of potential life lost due to premature mortality plus years of healthy life lost to disability). Estimates are based on relationships between mode of transport and injury risk together with the effects of changes in physical activity and air pollution on acute respiratory infections, breast cancer, cardio-respiratory and cardiovascular diseases, colorectal cancer, diabetes, dementia, and lung cancer. Savings depend on the method chosen for valuing deaths and DALYs. The authors found that, for the scenarios they tested, around 80–90% of the benefits arose from increased physical activity, with injury reduction contributing modestly and air pollution reduction only marginally [26]. However, the balance of benefits depends on the urban setting, including background levels of air pollution, physical activity, and so on. ITHIM has been adapted for New Zealand, and has been used to estimate the health benefits of an increase in active trips and a reduction in car trips [27].

Few other benefits of active travel interventions are likely to be of sufficient magnitude to make a material difference to benefit/cost ratios other than, potentially, savings from reduced congestion and noise. However, the latter are particularly specific to city, neighbourhood, and time. They have been estimated for Copenhagen at USD 0.073/km and 0.056/km, respectively [17], somewhat lower than cycling’s estimated social benefit from reduced road injury of USD 0.085/km. Average congestion and noise cost savings were also given in a cross-European study [28] at USD 0.055/km and 0.003/km, respectively. In relation to decongestion, because many more car trips take place in a city than active travel trips, a big proportional increase in active travel is needed to substantially reduce car trips and improve congestion. Moreover, where levels of congestion and noise are low compared with big cities, as in provincial cities, congestion and noise saving benefits will be small.

In short, a growing literature suggests, overall, that the benefits of active travel significantly exceed the costs, but few studies use empirical estimates based on controlled (or quasi-experimental) designs, and there are also limitations in design or scope of even the robust studies [21]. For example, climate benefits are seldom included. There are also important distinctions between what economists call ‘social’ (public) and private benefits/costs. Some studies identify a mix of private and social costs and benefits. Our view is that, in carrying out a CBA, private costs and benefits should not be counted as they are internalized in the decision to use a car or use active travel. Thus, the ‘baseline’ state before a CBA is performed is that a level of walking and cycling is taking place for which the benefits exceed the costs (‘consumer surplus’). In our study, we evaluated the social benefits (including external environmental benefits) arising from the *increased* active travel and compared those with the social (resource) costs of the infrastructure investment. Similarly, we took a national rather than local perspective while acknowledging that for the local governments appraising an investment in active travel infrastructure and programmes, some of the benefits are likely to accrue outside their jurisdiction and central government might not always recognise those spillovers with appropriate funding support.

## 2. Methods

### 2.1. Evaluating an Active Travel Intervention in the New Zealand Context

We set out to conduct an analysis that went beyond modelling of an active travel intervention to an empirical economic estimation of the net costs and benefits of an active travel intervention. ‘ACTIVE’ (Activating Communities to Improve Vitality and Equality) aimed to determine whether a combined central and local government-funded initiative to promote cycling and walking succeeded in altering travel patterns of residents in two North Island provincial cities, New Plymouth and Hastings (with populations of around 74,000 and 73,000, respectively, in 2013). The Model Communities Programme (MCP) aimed to deliver safe urban environments that would encourage ‘novice users’ to walk or cycle to school or to work in fully integrated walking and cycling transport networks [29]. New Plymouth District Council and Hastings District Council were selected based on the central government’s criteria. The MCP provided NZ$13.1 million (NZ$1~USD 0.73 at time of writing) spread across the two cities for building a mix of separated cycleways, walkways, and cycle lanes and associated education and promotion [30]. Infrastructure spending was $11.2 million, of which the New Zealand Transport Agency funded $7.8 million; education/promotion spending was $1.9 million.

Both cities used the MCP funding to extend walking and cycling networks. Hastings invested in four arterial paths (29.5 km), to link the city to surrounding centres, and connected these with more than 50 km of marked on- and off-road walking and cycling ‘collector’ facilities. To encourage the acceptance of cycling on the road, Hastings District Council also ran a substantial ‘Share the Road’ marketing campaign. New Plymouth, which already had an extensive network of tracks, invested much of the money on connecting and upgrading existing paths with the creation of an additional 12 km of off-road facilities and more than 20 km of on-road marked cycle lanes. Other interventions to make the city friendlier for walking and cycling included installing cycle parking, widening path entries, creating a number of shared spaces with reduced speed limits for vehicles (30 km/h), substantial media campaigns and events, and cycle-skills training at local schools [31].

The various aspects of the initiative were designed to be complementary and therefore more likely to be synergistic in effect [32]. Equity considerations were included: the New Zealand Transport Agency noted, with respect to the Hastings plan, that ‘An additional focus has been on disadvantaged communities where accessible transport options are important and the health benefits of active transport modes are the greatest’ [31] (p. 15).

We investigated whether the intervention moved travel away from motorised modes (mainly driving or being a passenger in a motor vehicle) and towards active modes while more generally increasing levels of physical activity. The details of the methods used have been described in Chapman et al. [10] and the main non-economic results in Keall et al. [9]. The central element of the quasi-experimental design was to match the two intervention cities with comparable control cities so that extraneous factors which might be changing (e.g., oil prices) could be excluded from an estimation of the effects of the initiative. Such controlled before-and-after studies at a population level are rare [18,33]. The two ‘control’ cities, Whanganui and Masterton, were smaller (populations 42,000 and 23,000, respectively, in 2013) but suitable to act as controls as they were broadly comparable to the intervention cities (physically close, similar climatic conditions, relatively similar transport characteristics, such as proportion cycling to work (New Plymouth 2.0%; Hastings 2.7%; Whanganui 3.3%; Masterton 2.8%); percentage walking or jogging to work (5.3, 4.0, 5.3, and 5.2); and percentage using public bus (0.4, 0.3, 0.3, and 0.3)) and the control cities were interested in encouraging active travel, but had not received additional central government funding for this purpose.

### 2.2. Appraising the Intervention Costs and Benefits

On the cost side, upfront costs (one-off investment and programme costs, rather than repeated costs) dominate, although we include maintenance costs. We take the upfront cost of the intervention as evenly spread between years 1 and 2 (2011 and 2012), with most of the core spending (NZ$13.1 million) going on infrastructure and the remainder on education and promotion. Another $17.1 million was spent by the two city councils on infrastructure, such as a central business district upgrade, but very little of this was to support active travel. In a sensitivity analysis, we examine the impact on results if some of this is assumed to have supported active travel.

The maintenance cost stream is estimated using two sources. One (NZ$0.185 million/year) is an *ex ante* cost estimate from the two district councils [34]. A second (NZ$0.16 million/year) is based on Sælensminde’s estimate that the annual maintenance cost was 1.47% of (one-off) construction costs [11]. The latter appears low considering Norway’s weather conditions are more extreme than New Zealand’s. We use the upper estimate (NZ$0.185 million/year).

The discount rate is a significant variable in most CBAs. Two values are used in order to represent a range of perspectives. The base value of the discount rate is taken as 3.5% real (excluding inflation), a choice aligned with O’Dea and Wren [35], who recommend 3.5% in the health sector, and with the U.K. Green Book [36]. To give a sensitivity range, we report results for 3.5% and 6% (the NZ Treasury rate for transportation investments).

The horizon chosen for this CBA is 20 years, on the basis that active travel infrastructure might need rebuilding beyond such a horizon. Note, however, that separated cycling tracks (as in one of the intervention cities) are likely to last longer, so this assumption could cause benefits to be underestimated. The CBA reference point is taken as 2010; the intervention (building cycle paths, etc.) began in financial year 2010/2011 (1 July 2010–30 June 2011), while the initial year for the survey was 2011 (too soon for any impacts to have occurred); follow-up surveys were taken in 2012 and 2013.

The main mode-related finding of the (non-economic) part of the study was that, relative to the control cities, the odds of trips being by active modes (walking or cycling) increased by 37% (95% CI 8% to 73%) in the intervention cities between baseline and post-intervention. The net proportion of trips made by active modes increased by about 30% relative to a background decline in active travel occurring in the control cities [9]. There was a decline in walking and cycling in the control cities in contrast to active transport being maintained in the intervention cities from year 2, 2012 (with a 15% relative increase), which reached a 30% relative increase level by year 3, 2013. The 30% difference in active transport rates and the small reduction in car use associated with the intervention are assumed to be stable from year 3 onwards. Note that the intervention was thus an example of what Hutton describes as ‘where environmental health interventions … hold gains already achieved, and prevent “back-sliding”, which is often not taken into account in evaluation’ [19] (p. 10).

The net 30% increase in active trips implies a 5.3% decrease (Table 1) in the relative number of motorized trips. All of the motorized trips replaced by active trips are assumed to be short trips (less than 5 km in length), averaging 2.2 km. The reduction in motorized trips equates to 1.21% of total (motorized) vehicle kilometres travelled (VKT). This reduction in car use of zero in 2011, 0.6% in 2012, and 1.21% (ongoing) by year 3 (2013) is a significant input into valuing carbon emission reductions.

Benefits to health are estimated from the changes in use of active modes. We use ITHIM for modelling the health benefits, which are expressed in avoided DALYs (avoided loss of years of life, from injury and avoided illness) and avoided deaths. The estimates are based on the estimated health gains which would accrue to New Plymouth and Hastings from the changes in active travel estimated. Our estimate is that the impact of the intervention on the population of these two cities is a reduction of 34.5 DALYs and two deaths, due largely to reductions in cardiovascular and inflammatory heart disease but also reductions in diabetes, depression, cancers and respiratory diseases, and injuries, for the cities in question.

To value the quantified health impacts, we use an estimate of the Value of a Statistical Life (VOSL) provided by the Ministry of Transport [37]. The VOSL (discussed in [15] and estimated at NZ$3.5 million in 2009 dollars) and the discount rate jointly determine the value of a statistical life *year* (VOSLY); for example, taking a 40-year horizon (remaining life years) and a discount rate of 3.5%, the VOSLY is NZ$164,000. The value of a DALY (a disability-adjusted life year lost) can be approximated by a VOSLY.

To value carbon emission reductions, we first assume that the reductions arise in proportion to VKT reductions (given above). We use a standard factor for CO_2_ emissions per km for a typical motor vehicle based on the average 0.2359 kg of CO_2_ per VKT for the passenger fleet. The value of carbon emission reductions is a controversial matter. One assumption is to take NZ$50 per tonne as the relevant unit value. This value is above the current price of traded CO_2_ units in the emission trading scheme (ETS), of around $20/tonne, but well below a value reflecting international estimates of the social cost of carbon (i.e., the estimated damage caused by a marginal tonne of CO_2_ emitted). The ETS value is, however, not useful as it is artificially constrained by political factors. A more reliable social cost of carbon value is US$125 per tonne of CO_2_ (currently NZ$178), which is based on van den Bergh and Botzen [38]. We use this as a best estimate [13], but also use NZ$50 in a sensitivity analysis.

One benefit of the intervention which could in principle be counted is the reduction in the cost of insecurity felt by cyclists and walkers as they travel along the edge of a road. This external cost can be saved by high-quality cycle lanes and footpaths. Saelensminde [11] estimates this cost in Norway at NOK 2 per km, or about NZ$0.34/km, or NZ$3.4–6.8/h. While this is potentially a significant benefit, we exclude it due to a lack of local confirmation; its exclusion suggests our results may underestimate benefit/cost ratios.

## 3. Results of the CBA

The key CBA result is that the costs are heavily outweighed by the benefits from investing in active travel at a discount rate of 3.5%. It remains so even in the sensitivity case of a discount rate of 6%. Reflecting this, benefit/cost ratios are around 11 to 1. These results are summarized in Table 2, in net present value (NPV) terms, in 2010 dollars. Focusing on benefits alone, health and injury avoidance benefits exceed the carbon emission reduction benefits estimated to arise from the active travel investment even with a relatively high unit value of carbon reductions (US$125/tonne).

A sensitivity analysis shows that if the unit value assumed for carbon dioxide savings is taken to be NZ$50/tonne of CO_2_, the present value of emission reductions falls to $0.7 million, and net benefits fall slightly, to NZ$149.3 million (at a 3.5% discount rate). The benefit/cost ratio is slightly reduced, at 10.9.

A further sensitivity test addresses the impact of changes in upfront costs. We examined the unlikely case that the active travel infrastructure investment would require complete renewal after 10 years rather than 20. This alternative assumption lowered the benefit/cost ratio from 11.1 to 8.3 (for a 3.5% discount rate and a NZ$178 carbon price). That is, even if active travel infrastructure must be renewed more often than expected, it would remain a worthwhile investment under most conditions.

An additional sensitivity test examined the effect of assuming that one quarter of additional upfront costs incurred by local councils were for supporting active travel. In this case the benefit/cost ratio fell to 8.6 (for a 3.5% discount rate and a NZ$178 carbon price). 

A last sensitivity test allowed for the possibility that the mode shift impact might decay gradually over time. When we tested for a 5% per year reduction in the profile of benefits after year 3, the benefit/cost ratio was reduced to 7.8 (for a 3.5% discount rate and a NZ$178 carbon price).

## 4. Discussion

This study has focused on the health (including injury) and carbon reduction benefits of increased active travel in two provincial cities (with, together, over 3% of national population). It is based on a quasi-experimental study of a walking and cycling infrastructure investment and promotion programme focused on investment in improving urban active travel networks. One of the two intervention cities added 30 km of arterial paths, 50 km of on- and off-road walking and cycling facilities, and a ‘Share the Road’ campaign. The other added 12 km of off-road facilities, 20 km of cycle lanes, cycle parking, ‘shared spaces’, and ran media campaigns, events, and cycle-skills training.

The study is one of the few cost-benefit studies to date of the results of an intervention already implemented. It is also one of the few instances in which ITHIM has been applied to a completed experimental study.

Leaving other benefits aside, health (predominantly) and carbon emission benefits fully justify the investment in active travel. The benefit/cost ratios were found to be around 11:1. This ratio was little reduced when we applied a lower price of NZ$50 for carbon dioxide emissions (the ‘social cost of carbon’). A higher discount rate also led to a slightly lower benefit/cost ratio. In addition, the benefit/cost ratio was not substantially reduced in testing of other plausible sensitivity scenarios such as those relating to infrastructure renewal expenditure and the decay of the mode shift effect over time.

As noted earlier, the impact of health gains, injury reductions, and carbon emission reductions are together likely to be much more important than congestion and noise benefits. In these provincial cities, congestion and noise are modest and so are excluded from the study. A more comprehensive study of modal change in a bigger city would, preferably, measure these effects, and provide an even better basis for allocating scarce transport resources.

How cyclists think about health effects is also important. Some writers [39] assume that the personal health benefits of cycling are reflected in the demand for cycling (and likewise for walking). This means the time cost (opportunity cost) facing cyclists is lower, they argue, because the health benefits are (already) taken into account. The implication is that there could be double counting if the health benefit were separately estimated and included in a CBA evaluating the benefits of an active transport intervention. However, while cyclists may be aware of private health benefits (just as they may be aware of a time penalty of cycling), the extent of these benefits is unlikely to be fully appreciated by cyclists and may well be significantly underestimated, in large part because the social health benefits (e.g., the benefits to the health system of better individual health) are unclear to them. This is in contrast to matters such as savings in motor vehicle operating costs, which are more likely to be built into the demand for active transport. Moreover, other co-benefits of cycling, such as decongestion of roads, or climate change mitigation benefits, are straightforward external benefits, and issues of possible double counting do not arise.

CBA is not a precise science and we agree with a number of authors of transport studies that it is not necessarily the optimal framework for prior appraisal or *ex post* evaluation of investments in active transport [14]. Multi-criteria analysis (MCA), for example, may well be a better decision-supporting framework than CBA for appraising ‘small schemes involving active travel interventions where the majority of benefits are very difficult to quantify—let alone monetize—but nevertheless are recognized to exist’ [14]. Authors of the Copenhagen study of cycling and car investments emphasise the ‘imperfect and conservative’ nature of CBA frameworks (p. 111) in reaching their conclusion that ‘there is a clear case to be made for infrastructural change in favour of cycling’ [17]. Nevertheless, they argue for the use of CBA in helping develop a political consensus.

The present study did not have the opportunity to test an alternative framework, such as MCA, to appraise beforehand the views of the ‘model communities’ involved in this study. Moreover, it may be that a CBA evaluation would have greater credibility in the transport setting, since it is more familiar than an alternative approach. We chose to focus our analysis on aspects of the CBA process that require careful consideration and sensitivity analysis. We also chose to underline the importance of the co-benefits associated with greater cycling levels and the avoidance of a decline in rates of cycling. As the CBA demonstrates, these are predominantly health related, but not entirely so.

The current study has shown a high benefit/cost ratio despite a conservative approach to valuing benefits. Moreover, several sensitivity tests did not alter the overall conclusions. However, as with all empirical studies carried out in particular cities, a limitation of this study is that behaviour in the two cities involved in the study may not be generalizable to other cities, or to other sorts of active travel interventions. Indeed, we know that provincial cities in New Zealand have generally been undergoing a drop in active travel while large cities have not. Nevertheless, this study does, we believe, tell us something that can be generalized, i.e., that when a concerted (and fittingly sized) active travel intervention is made in a city, there is likely to be a measurable, positive economic return on that intervention once the important benefits are taken into account.

## 5. Conclusions

In conclusion, the present cost benefit study has focused mainly on what are likely to be the most important benefits and costs relating to an active travel intervention programme in order to provide what few other studies have done, namely, an estimate in economic terms of the value of such a programme. Some key parameters have been varied in sensitivity analyses, but the primary conclusion remains robust, that such an intervention programme is likely to be economically worthwhile. The benefit/cost ratio of the programme (over 10:1) is well in the range to justify the investment involved, taking into account health and injury savings and the value of carbon emission reductions.

## Figures and Tables

**Table 1:** Summary of ACTIVE results underlying the cost benefit analysis (CBA) calculations below.

Variable	Estimate
Net increase in non-motorized (active) trips (by 2013, in New Plymouth and Hastings)	30%
Increase in number of non-motorized (active) trips	17.3 million
Decrease in motorized trips	5.3% *
Saving in motorized vehicle-km (VKT) as % of total motorised VKT	1.21%
Saving in annual motorized VKT	4.87 million
Saving in CO_2_ emissions	1149 tonnes **
Health benefit (per year)	34.5 DALYs; 2 deaths saved

* One non-motorized trip saves one motorized trip but only 15% of trips are non-motorized. ** Using standard emissions factor for the passenger vehicle fleet of 0.2359 kgCO_2_/vehicle kilometre travelled (VKT). DALY = disability-adjusted life year.

**Table 2:** Costs and benefits (in millions in net present value (NPV) terms) of an intervention to increase cycling and walking.

Discount Rate	Costs *	Health and Injury Benefits	CO_2_ Reduction Benefits	Net Benefits	Benefit/Cost Ratio
3.5%	$15.0 million	$163.6 million	$2.6 million	$151.2 million	11.1
6.0%	$14.1 million	$153.1 million	$2.1 million	$141.1 million	11.0

* Costs include upfront and ongoing (maintenance) costs, converted to NPV terms. NB: Value of carbon dioxide emission reductions is taken to be US$125/tonne of CO_2_ (NZ$178/tCO_2_).
